# The complete mitogenome of the large toothed toad, *Oreolalax major* (Anura: Megophryidae) with phylogenetic analysis

**DOI:** 10.1080/23802359.2020.1726223

**Published:** 2020-02-11

**Authors:** Tao Huang, Lin Cui, Diyan Li, Xiaolan Fan, Mingyao Yang, Deying Yang, Qingyong Ni, Yan Li, Yongfang Yao, Huailiang Xu, Bo Zeng, Ying Li, Feida Sun, Mingwang Zhang

**Affiliations:** a College of Animal Science and Technology, Sichuan Agricultural University, Chengdu, PR China;; b Animal Genetic Resources Exploration and Innovation Key Laboratory of Sichuan Province, Sichuan Agricultural University, Chengdu, China;; c College of Life Science, Sichuan Agricultural University, Ya’an, PR China

**Keywords:** Complete mitogenome, *Oreolalax major*, phylogenetic analysis

## Abstract

The complete mitochondrial genome of the *Oreolalax major* (17,786 bp long) was obtained in this study. It includes 13 protein-coding genes (PCG), two ribosomal RNA (rRNA) genes, and 23 transfer RNA (tRNA) genes (GenBank accession number MN803320). The phylogenetic tree indicates that the *O. major* is closely related to the *O. xiangchengensis*.

The large toothed toad (*Oreolalax major*), belonging to the family Megophryidae, ranges in montane regions of southern Gansu (Wenxian County) and central Sichuan (Beichuan, Baoxing, Emei, Dujiangyan, Hongya, Luding, Pingshan and Wenchuan counties), China (Fei et al. 2012; Frost 2020). The near complete mitogenome of *O. major* has been reported by (Liu et al. 2017). Here, we further provide a complete mitogenome of *O. major*, which helps to further understand the information of *O. major* and explore the phylogenetic relationship among the genera of Megophryidae.

The sample of *O. major* was obtained from Wawushan Nature Reserve (29°34′26.72″N, 102°56′35.73″E., elev. 1394 m), Sichuan Province, China, and the specimen was deposited in the Museum of Sichuan Agricultural University (Specimen voucher: 20120256). We extracted the total DNA from ethanol-preserved muscle tissue using the Ezup pillar genomic DNA extraction kit (Sangon Biotech, Shanghai, China). DNA sample was sent to Personal Biotechnology (Shanghai, China) for library construction and sequencing on an Illumina MiSeq platform (PE400).

The new *O*. *major* mitogenome (MN803320) sequence is 17,786 bp long and contains 13 protein coding genes (PCGs), two ribosomal RNA (rRNA) genes, 23 tRNA genes (including an extra *trnM* gene) and a control region (D-loop). The base composition of the mitogenome is 28.75% A, 14.31% G, 24.31% C, 32.63% T. The mitogenome structure of *O. major* is similar to that of other Megophryidae (Xiang 2013; Liang 2016; Liang et al. 2016). Eight *tRNA* (*tRNA-Gln*, *Ala*, *Asn*, *Cys*, *Tyr*, *Ser*, *Glu*, and *Pro*) and the *nad6* genes are encoded on the L-strand, and the rest of genes are encoded on the H-strand. Among the 13 PCGs, the start codon of the *nad3* is ATA, three PCGS (*cox1*, *cox2*, and *nad6*) initiate with GTG, and the rest nine PGCs use ATG. For the terminal codon, three PCGs (*cox1*, *nad5* and *nad6*) use AGG as stop codon, and three (*nad2*, *atp8* and *nad4l*) ended with TAA. Two PCGs (*nad1* and *atp6*) ended with TA, whereas four (*cox2*, *cox3*, *nad4* and *cytb*) ended with incomplete stop codon (T-). The two rRNA genes (12S and 16S) are 937 and 1,579 bp in length, respectively. Within the genus of *Oreolalax*, the *O. rhodostigmatus* mitogenome was the largest, at 18,676 bp, followed by *O*. *jingdongensis* (17,864 bp), *O*. *major* (17,786 bp), *O*. *lichuanensis* (17,702), *O*. *multipunctatus* (17,358 bp), and *O*. *xiangchengensis* (17,110 bp). Comparing the intraspecific mitogenome size variation in this toad, the mitogenome of *O*. *major* (17,786 bp) from Wawushan in this study is larger than that (17,431 bp) from Luding reported by Liang et al. (2016). The differences in mitogenome size was due to the size variation in control region.

We used MEGA7.0 (Kumar et al. 2016) to reconstruct the phylogenetic tree of the family Megophryidae. The final alignment consisted of 21 species from seven genera (*Oreolalax, Leptobrachium, Scutiger, Leptolalax, Megophrys, Pelodytes,* and *Xenopus*). Phylogenetic trees based on these mitochondrial genomes show that *O. major* was the sister taxon to *O. xiangchengensis* (Figure 1). This complete mitogenome would contribute to further investigations of molecular evolution and conservation of genus *Oreolalax*.

**Figure 1.**
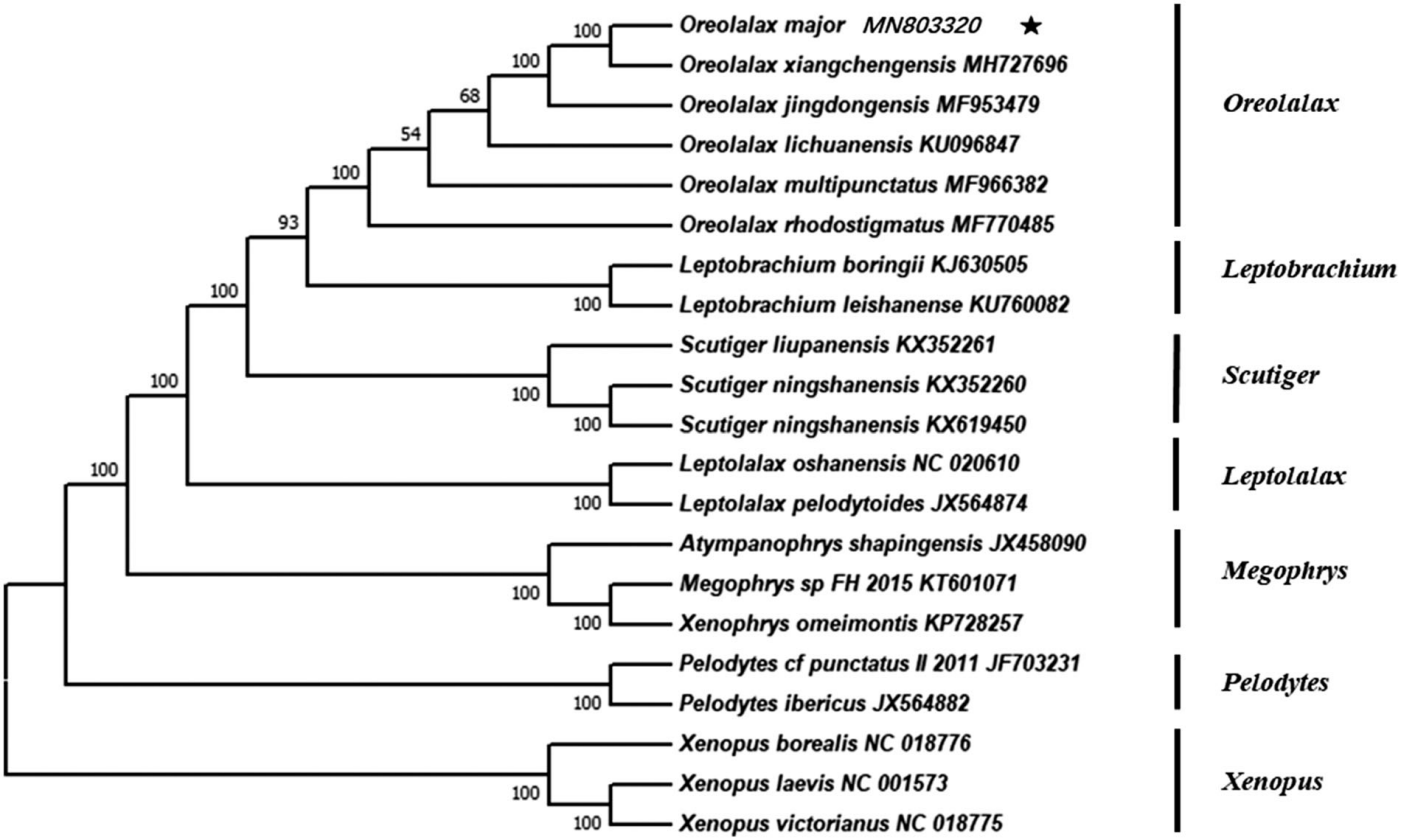
Neighbour-joining phylogenetic tree of family Megophryidae was built on mitogenomic sequences of all 13 combined protein-coding genes from 21 species. Bootstrap values are shown in the middle of the branches connected to the nodes.
